# Radiation Exposure in Paediatric Orthopaedic Trauma Surgery

**DOI:** 10.7759/cureus.96053

**Published:** 2025-11-04

**Authors:** Kraig Jamieson, Simon Barker

**Affiliations:** 1 Ophthalmology, Ninewells Hospital, Dundee, GBR; 2 Paediatric Orthopaedics, Royal Aberdeen Children's Hospital, Aberdeen, GBR

**Keywords:** diagnostic reference levels, fluoroscopy, orthopaedic procedures, paediatric orthopaedics, radiation exposure

## Abstract

Introduction: Fluoroscopy is widely used in paediatric orthopaedic surgery; however, national diagnostic reference levels (DRLs) for intraoperative radiation exposure are lacking. Establishing local DRLs is essential to benchmark practice, optimise patient safety, and inform future national standards. This study quantifies radiation exposure across common paediatric orthopaedic procedures to provide data for local reference ranges.

Methods: A retrospective observational study was performed at a single-centre NHS specialist children’s hospital, including 480 procedures in patients aged <18 years over a four-year period. Procedures were categorised by anatomical region (elbow, ankle, pelvis) and type: manipulation under anaesthesia (MUA), MUA with K-wire fixation, and open reduction and internal fixation (ORIF). Fluoroscopy radiation exposure (mGy/cm²) was recorded. Descriptive statistics (25th percentile, median, 75th percentile, mean) were calculated, and differences between procedure types were assessed using Mann-Whitney U tests (p < 0.05). Radiation doses were contextualised against local background exposure and transatlantic flight equivalents.

Results: Slipped upper femoral epiphysis (SUFE) pinning had the highest mean exposure (384.5 mGy/cm²). In the elbow subgroup, ORIF had the highest mean exposure (13.9 mGy/cm²), while MUA + K-wire fixation had the highest ankle exposure (12.4 mGy/cm²). MUA + K-wire procedures showed significantly higher exposures than simple MUA in both the elbow (p < 0.001) and ankle (p < 0.001). Pelvic MUA procedures had higher exposure than elbow or ankle MUA (p < 0.001).

Conclusions: This study establishes the first detailed local reference ranges for intra-operative fluoroscopy in paediatric orthopaedics. Findings demonstrate procedure-specific variability in radiation exposure, support the development of local DRLs, and provide a benchmark for safer practice and future national standardisation.

## Introduction

Fluoroscopy is a real-time X-ray imaging technique widely used in orthopaedic surgery to visualise bony anatomy during surgical procedures. This enables intra-operative optimisation of fracture treatment, localisation of pathology, and implant placement, potentially reducing operating times and patient morbidity [1]. Paediatric orthopaedic surgeons are among the most prolific users of fluoroscopy, yet little research has been devoted to dose exposure within the paediatric setting [2].

Ionising radiation carries well-established risks, including an increased likelihood of malignancy, though this is difficult to accurately quantify due to the multifactorial nature of cancer aetiology. Research indicates that children are particularly radiosensitive [2-7]. This is believed to be due to their smaller body mass and rapidly dividing cells, which render them more vulnerable to radiation effects [2-7]. While single X-ray exposures are typically low, complex cases or long-term conditions requiring repeated imaging, such as patients with cerebral palsy or developmental hip disorders, can result in significant cumulative lifetime exposure. Quantifying radiation exposure in paediatric orthopaedic procedures is therefore critical for informed consent and patient safety [8].

In the United Kingdom, the Ionising Radiation (Medical Exposure) Regulations (IR(ME)R, 2017) establish legal requirements to minimise unnecessary radiation exposure [9]. These regulations stipulate that patients should receive only the minimum radiation necessary for clinical purposes, in accordance with the As Low As Reasonably Achievable (ALARA) principle. For quality assurance, IR(ME)R recommends establishing diagnostic reference levels (DRLs), which serve as benchmarks indicating expected radiation doses for specific procedures [10]. Ideally, national DRLs would be available for all procedures, where absent, local DRLs based on institutional audits and literature reviews are encouraged [11,12].

Despite the routine collection of radiation exposure data within the NHS, no detailed assessment to deliver DRLs has been undertaken in paediatric orthopaedic surgery. Previous studies have examined intra-operative radiation, but most focus on adults and include only small paediatric subsets [2,13-15]. Pillai et al. developed local DRLs for selected paediatric procedures in Scotland, but their cohort included only children aged 5-10 years and assessed a few procedures [2]. Other studies, including Kropelnicki, Giordano, and Rashid, provide comparative radiation data but often combine adult and paediatric populations or report limited procedural breakdowns [13-15]. Therefore, a comprehensive evaluation of radiation exposure across common paediatric orthopaedic procedures remains lacking.

This study addresses this information gap by analysing intra-operative radiation exposure for paediatric orthopaedic procedures at a large single-centre specialised children’s hospital. Procedures were examined across multiple anatomical regions, the elbow, ankle, and pelvis, to establish local reference ranges, including the 25th percentile, median, 75th percentile, and mean. Radiation doses were contextualised against local background radiation and other common sources of exposure, providing clinicians with a practical, patient-centred framework to interpret risk [16-20]. By quantifying procedure-specific exposure, this study aims to support safer fluoroscopic practice, optimise patient care, and provide a foundation for local DRLs [8,19,20].

Primary objectives

This study aims to quantify intra-operative radiation exposure in paediatric orthopaedic procedures performed at a single-centre NHS specialist children’s hospital, specifically for fracture manipulation under anaesthesia (MUA), MUA with K-wire fixation, and open reduction and internal fixation (ORIF) across three anatomical regions: elbow, ankle, and pelvis.

Secondary objectives

This study aims to establish local reference ranges (25th percentile, median, 75th percentile, and mean) for radiation exposure; to contextualise these values against local background radiation and estimated transatlantic flight equivalents; and to benchmark findings against published literature to identify opportunities for dose optimisation.

This study provides a foundation for local DRLs, supports evidence-based optimisation of fluoroscopic practice, and, by informing clinicians, aims to improve patient safety in paediatric orthopaedic surgery. The results may also guide future research into cumulative radiation exposure in children with long-term orthopaedic conditions, enhancing understanding of procedure-specific radiation risks in this vulnerable population.

## Materials and methods

This retrospective observational study was conducted at a single-centre NHS specialist children’s hospital and included paediatric orthopaedic procedures performed between 1 September 2018 and 1 September 2022. All patients aged under 18 years who underwent operations requiring intra-operative mobile fluoroscopy were considered eligible. Fluoroscopy dose-area product (DAP, mGy/cm²) values were automatically recorded by the image intensifier and archived within electronic patient records. These were cross-referenced with operative notes to identify the corresponding procedure and site of operation.

Procedures were categorised by anatomical region (elbow, ankle, pelvis) and type: MUA, MUA with K-wire fixation, and ORIF. To reduce errors due to a small sample size, procedures where fewer than 10 cases had been undertaken in the study period were excluded.

For each procedure, descriptive statistics were calculated, including the 25th percentile, median, 75th percentile, and mean radiation exposures, following previously published methods for developing DRLs in paediatric orthopaedics. Comparative analyses were conducted using the Mann-Whitney U test, with statistical significance set at p < 0.05. Analyses were performed using IBM SPSS Statistics for Windows, Version 26 (Released 2018; IBM Corp., Armonk, New York, United States). To provide clinical relevance, estimated relative risks were contextualised by comparing intra-operative exposures with local background radiation levels and equivalent exposures from transatlantic flights [16-18].

This study was classified as a service evaluation and conducted in accordance with local governance policies and regulations. Formal NHS Research Ethics Committee approval was not required. All patients (or their guardians) provided written informed consent for the use of intra-operative fluoroscopy as part of their surgical procedure prior to the service evaluation. Only routinely collected, anonymised data were used for analysis, and no identifiable patient information was collected or assessed. Importantly, DAP (mGy/cm^2^) provides an objective measure of the total radiation output from the fluoroscopic system but does not directly represent the absorbed or effective dose received by the patient. The biological dose depends on several factors, including beam geometry, tissue attenuation, and patient anatomy, which could not be accounted for in this study.

## Results

A total of 480 procedures were analysed across three anatomical regions: elbow (five procedures), ankle (five procedures), and pelvis (six procedures) (Table 1). For each procedure, the 25th percentile, median, 75th percentile, and mean radiation exposures were calculated.

**Table 1:** Reference ranges for intra-operative fluoroscopy exposure by procedure and anatomical site MUA: manipulation under anaesthesia; ORIF: open reduction and internal fixation; SUFE: slipped upper femoral epiphysis; TENS: titanium elastic nailing system Reference ranges for intra-operative fluoroscopy exposure (mGy/cm²) for each procedure analysed in this study. Procedures are grouped by anatomical region (elbow, ankle, pelvis). Data include the number of cases (n), 25th percentile, median, 75th percentile, mean, and interquartile range (IQR). Procedures with fewer than 10 cases were excluded

Procedure	Number of cases (n)	25^th^ percentile	Median	75^th^ percentile	Mean	Interquartile range
Elbow						
MUA	42	1.2	2.2	4.6	2.8	3.4
ORIF	44	5.4	12.6	18.6	13.9	13.2
TENS nailing	13	4.2	7.6	10.0	7.8	5.8
MUA + K-wire	113	4.6	7.7	12.7	9.9	8.1
Ankle						
MUA	16	0.9	1.8	5.0	2.7	4.1
Injection	15	0.9	1.3	1.9	1.5	1.0
Osteotomy	23	1.2	4.4	12.4	7.2	11.2
ORIF	39	3.7	8.3	13.0	10.3	9.3
MUA + K-wire	12	4.4	8.8	21.3	12.4	16.9
Pelvis						
ORIF (plate)	28	13.4	44.4	64.5	74.0	51.1
ORIF (screw/nail)	19	22.9	79.9	218.8	111.8	195.9
SUFE pinning	16	127.5	210.7	516.2	384.5	388.7
Injection or biopsy	26	5.1	9.2	21.6	17.9	16.5
Metal removal	12	5.3	16.4	26.1	17.8	20.8
EUA	48	6.3	11.6	19.4	19.0	13.1
Osteotomy	14	8.1	24.2	36.9	28.5	28.8

Slipped upper femoral epiphysis (SUFE) pinning had the highest mean exposure of any procedure (384.5 mGy/cm², n = 16), while ankle injection had the lowest (1.5 mGy/cm², n = 15). Within the pelvis group, injection or biopsy procedures also showed low exposure (mean 5.1 mGy/cm², n = 26).

In the elbow subgroup, MUA + K-wire fixation was the most common procedure (n = 113) with a mean exposure of 9.9 mGy/cm², while ORIF demonstrated the highest exposure (13.9 mGy/cm², n = 44). Exposure from MUA + K-wire was significantly greater than simple MUA (2.8 mGy/cm², p < 0.001). A similar trend was observed in the ankle, where MUA + K-wire (12.4 mGy/cm²) had significantly higher exposure than MUA alone (2.7 mGy/cm², p < 0.001). ORIF of the elbow also carried significantly higher exposure than MUA + K-wire (p = 0.026).

Comparisons across anatomical regions demonstrated no significant difference between MUA of the elbow and ankle (p = 0.677). However, both were significantly lower than MUA of the pelvis (19.1 mGy/cm², p < 0.001).

To provide clinical context, mean exposures were compared with typical UK background radiation (2.7 mSv/year) and equivalent doses from a transatlantic flight (Table 2) [16,18]. This demonstrated that some procedures, such as SUFE pinning, equated to over a lifetime of natural background exposure, while minor interventions corresponded to less than a year. Importantly, comparisons to local background radiation and transatlantic flight exposure are provided for illustrative context only. DAP (mGy/cm^2^) represents the total energy delivered to a given area, and it cannot be directly converted to effective dose without patient and projection-specific conversion factors. Therefore, these comparisons should be interpreted cautiously and not as direct estimates of biological dose.

**Table 2:** Comparison of mean radiation exposure per procedure with common radiation benchmarks EUA: examination under anaesthesia; ORIF: open reduction and internal fixation; MUA: manipulation under anaesthesia; SUFE: slipped upper femoral epiphysis For reference, the number of transatlantic flights (0.08 mSv per flight) and years of living in the UK (2.7 mSv per year) correspond to the cumulative exposure required to equal the mean radiation of each procedure. Data sources: [16,18]

Procedure	Mean radiation exposure (mGy/cm^2^)	Number of transatlantic flights (0.08 mSv per flight) [18]	Years of living in the UK (2.7 mSv per year) [16]
SUFE pinning	384.5	4807	142.4
Pelvic ORIF (screw/nail)	111.8	1397	41.4
Pelvic ORIF (plate)	74.0	925	27.4
Osteotomy	28.5	317	10.6
EUA	19.0	238	7.1
Injection or biopsy	17.9	223	6.6
Metal removal	17.8	222	6.6
ORIF elbow	13.9	174	5.2
MUA + K-wire foot & ankle	12.4	155	4.6
ORIF foot & ankle	10.3	129	3.8
MUA + K-wire elbow	9.9	124	3.7
TENS nailing forearm	7.8	97	2.9
Osteotomy foot & ankle	7.3	91	2.7
MUA elbow	2.8	36	1.1
MUA ankle	2.7	34	1.0
Injection ankle	1.5	18.5	0.6

## Discussion

This study provides one of the largest single-centre evaluations of intra-operative fluoroscopy exposure in paediatric orthopaedics, establishing local reference ranges across multiple procedures and anatomical regions. Significant variability in radiation dose between procedures was clearly demonstrated, with higher exposures seen in fixation compared with manipulation alone, reflecting the relatively increased complexity of procedures involving fixation and therefore the need for increased fluoroscopy. Similarly, pelvic procedures showed increased reliance on imaging compared with procedures involving the extremities.

The interquartile ranges were dramatically greater in the pelvic procedure group, highlighting high case-to-case and potentially practitioner-to-practitioner variability (though the latter was not formally assessed in this study). These findings offer a practical framework for benchmarking fluoroscopy use and support the development of local DRLs.

Comparisons with published data (Tables 3-5) highlight the importance of paediatric-specific DRLs. Table 3 shows that the 75th percentile exposures for MUA of the elbow and ankle exceed those reported by Pillai et al., likely reflecting inclusion of older children and more complex cases in this study [2]. Lower 25th percentile and median values suggest greater variability locally. Table 4 demonstrates that adult or mixed cohorts in Kropelnicki et al. and Giordano et al. may underestimate paediatric exposures [13,14]. Median exposures from Rashid et al. (Table 5) were consistently lower than our findings, underscoring the need for local paediatric reference ranges [15].

**Table 3:** Comparison of reference ranges for intra-operative fluoroscopy exposure with Pillai et al. [2] MUA: manipulation under anaesthesia Reference ranges (25th percentile, median, 75th percentile) for elbow and ankle MUA procedures in this study compared with values reported by Pillai et al. [2]. Values are in mGy/cm², with the number of cases (n) indicated in parentheses

Anatomical region	25^th^ percentile (mGy/cm^2^)		Median (mGy/cm^2^)		75^th^ percentile (mGy/cm^2^)	
	This study	Pillai et al. [2]	This study	Pillai et al. [2]	This study	Pillai et al. [2]
Elbow MUA	1.2 (n = 42)	0.6 (n = 153)	2.2 (n = 42)	1.2 (n = 153)	4.6 (n = 42)	2.4 (n = 153)
Ankle MUA	0.9 (n = 42)	1.7 (n = 56)	1.8 (n = 42)	3.1 (n = 56)	5.0 (n = 39)	4.6 (n = 56)

**Table 4:** Comparison of 75th percentile fluoroscopy exposure with Kropelnicki et al. [13] and Giordano et al. [14] MUA: manipulation under anaesthesia; ORIF: open reduction and internal fixation Comparison of 75th percentile radiation exposure (mGy/cm²) for selected procedures in this study with values reported by Kropelnicki et al. and Giordano et al. [13,14]. Values include the number of cases (n)

Anatomical area	This study (mGy/cm^2^)	Kropelnicki et al. [13] (mGy/cm^2^)	Giordano et al. [14] (mGy/cm^2^)
Elbow versus MUA + k-wire	12.7 (n = 113)	14.6 (n = 107)	
Ankle ORIF	13.0 (n = 39)	8.6 (n = 328)	11.9 (n = 46)
Pelvis ORIF (plate)	44.4 (n = 28)		84.8 (n = 18)
Pelvis ORIF (screw/nail)	79.9 (n = 19)		Screw: 77.4 (n = 29), nail: 66.1 (n = 98)

**Table 5:** Comparison of median fluoroscopy exposure with Rashid et al. [15] MUA: manipulation under anaesthesia; ORIF: open reduction and internal fixation Median radiation exposure (mGy/cm²) for selected paediatric orthopaedic procedures in this study compared with values reported by Rashid et al. [15]. Values include the number of cases (n)

Procedure	This study (mGy/cm^2^)	Rashid et al. [15](mGy/cm^2^)
Elbow		
MUA	2.2 (n = 42)	33.9 (n = 12)
ORIF	12.6 (n = 44)	81.3 (n = 12)
MUA + K-wire	7.7 (n = 113)	87 (n = 13)
Ankle		
MUA	1.8 (n = 16)	47.3 (n = 16)
ORIF	8.3 (n = 39)	37.4 (n = 22)

Some limitations should be acknowledged. The retrospective design relied on routinely collected exposure data, which may be influenced by patient body size, surgeon experience, or intra-operative complexity. Procedures with fewer than 10 cases were excluded, potentially omitting uncommon but clinically relevant exposures. The study also did not assess cumulative doses in patients undergoing repeated imaging, which may be clinically significant in conditions such as cerebral palsy.

These findings have practical implications. Establishing local DRLs allows clinicians to identify procedures associated with higher-than-expected exposures and provides a benchmark for self-reflective learning and quality improvement. Radiation reduction can be achieved through consistent use of pulsed or low-dose settings, optimising image intensifier positioning, and reinforcing training in ALARA principles. Regular audit against DRLs will further ensure safe practice [9].

Future research should investigate cumulative radiation exposure in children with chronic orthopaedic conditions and assess whether targeted interventions, such as simulation-based fluoroscopy training or standardised operative protocols, can meaningfully reduce exposure. Such work would contribute towards the eventual establishment of national paediatric DRLs and improved radiation safety in orthopaedic practice [9].

## Conclusions

This study provides local reference ranges for intra-operative fluoroscopy in paediatric orthopaedic procedures, highlighting variability in exposure across anatomical regions and surgical techniques. These findings highlight opportunities to establish local DRLs, support safer practice, and guide ongoing audit in line with IR(ME)R (2017). Future work should focus on cumulative exposure in children with chronic conditions to further inform risk-benefit discussions with patients and families.
